# Predicting recurrence of depression using cardiac complexity in individuals tapering antidepressants

**DOI:** 10.1038/s41398-023-02474-7

**Published:** 2023-05-30

**Authors:** Sandip V. George, Yoram K. Kunkels, Arnout Smit, Marieke Wichers, Evelien Snippe, Arie M. van Roon, Harriëtte Riese

**Affiliations:** 1 Department of Psychiatry, Interdisciplinary Center Psychopathology and Emotion Regulation (ICPE), University of Groningen, University Medical Center Groningen, Groningen, The Netherlands; 2 Department of Computer Science, University College London, London, UK; 3 Department of Vascular Medicine, University of Groningen, University Medical Center Groningen, Groningen, The Netherlands

**Keywords:** Depression, Predictive markers

## Abstract

It is currently unknown whether the complexity and variability of cardiac dynamics predicts future depression and whether within-subject change herein precedes the recurrence of depression. We tested this in an innovative repeated single-subject study in individuals who had a history of depression and were tapering their antidepressants. In 50 individuals, electrocardiogram (ECG) derived Interbeat-interval (IBI) time-series data were collected for 5 min every morning and evening, for 4 months. Usable data were obtained from 14 participants who experienced a transition (i.e., a clinically significant increase in depressive symptoms) and 14 who did not. The mean, standard deviation, Higuchi dimension and multiscale entropy, calculated from IBIs, were examined for time trends. These quantifiers were also averaged over a baseline period and compared between the groups. No consistent trends were observed in any quantifier before increases in depressive symptoms within individuals. The entropy baseline levels significantly differed between the two groups (morning: *P* value < 0.001, Cohen’s *d* = −2.185; evening: *P* value < 0.001, Cohen’s *d* = −1.797) and predicted the recurrence of depressive symptoms, in the current sample. Moreover, higher mean IBIs and Higuchi dimensions were observed in individuals who experienced transitions. While we found little evidence to support the existence of within- individual warning signals in IBI time-series data preceding an upcoming depressive transition, our results indicate that individuals who taper antidepressants and showed lower entropy of cardiac dynamics exhibited a higher chance of recurrence of depression. Hence, entropy could be a potential digital phenotype for assessing the risk of recurrence of depression in the short term while tapering antidepressants.

## Introduction

Determining the risk of recurrence of depression, especially when tapering antidepressants is a challenging problem. Tapering of antidepressants can typically lead to a worsening of depressive symptoms [1–4]. Hence, warning signs indicating the possibility of recurrence of depression or worsening of symptoms are of immense importance. Complex dynamical systems theory predicts the presence of early warning signals in the response of a system before many kinds of transitions [5]. Recent studies based on this have shown promise in predicting depressive episodes from momentary affect data [6–8]. Such changes in the dynamics of depression could lead to potential warning signals in cardiac dynamics as well. These could include measures such as heart rate, heart rate variability, and complexity, all of which have been shown to be altered in patients with depression [9–11].

Since the response of the heart is well understood to be nonlinear, it is prudent to study its nonlinear dynamics when seeking warning signals for depression [12]. These nonlinear dynamics can be quantified from the electrocardiogram (ECG) derived InterBeat-Interval (IBI) time series, using complexity measures such as the entropies, dimensions, and Lyapunov exponents. Deviations from healthy values for many of these measures, such as the fractal dimension, are associated with pathology [13, 14]. Disorders of various types, including mental disorders [15–21], are associated with a reduction in the complexity of dynamics of the heart. The complexity of cardiac dynamics, as well as simpler measures such as the mean and variability of IBI have been shown to be reduced in individuals diagnosed with depression, as well as dysphoria [15, 16, 22–24], although there is debate about whether this reduction can be explained completely by the effect of antidepressants [11, 25, 26]. For complexity measures of IBI time series to be potentially used as an early warning indicator for upcoming increases in depressive symptoms, a reduction in the complexity of cardiac dynamics must occur in the period before transitions towards higher levels of depression. This has not been empirically studied yet.

To examine whether a reduction in the complexity of cardiac dynamics over time occurs just before patients transition towards higher levels of depressive symptoms, a single-subject design including IBI time-series data may be employed. A between-subject design, on the other hand, is appropriate if one wants to study average differences in the complexity of cardiac dynamics that exist in the sample. The present TRANSitions In Depression (TRANS-ID) Tapering study employs a repeated single-subject design, where intensive longitudinal data of different types (momentary affect, physical activity and ECG) were collected for four months within formerly depressed individuals tapering their antidepressants, offering the possibility for both within-subject as well as between-subject studies [27–30].

We examine whether a decrease in the mean, standard deviation, and complexity in IBI time-series data as captured with the Higuchi dimension and multiscale entropy precedes a depressive transition (i.e., recurrence of depressive symptoms) by 4–8 weeks, a timescale observed in previous studies [6, 7, 31]. These complexity measures were chosen as they capture two different aspects of complexity. Fractal dimensions quantify long-range correlations in data, which are closely related to deviations from normal physiological regulation [14]. While several types of estimates of the fractal dimension exist [32, 33], in this work, we choose the Higuchi fractal dimension, a measure that has been extensively used to study cardiac data [34]. The multiscale entropy captures the information content in the time series across different time scales. We conduct repeated single within-subject analyses, where we study whether decreases in these quantifiers over time precede a transition towards depression for each individual separately in formerly depressed individuals who taper their antidepressant medication. Furthermore, to study average tendencies, we also conduct a between-subject analysis to test whether the baseline complexity (chosen as the first 4 weeks of assessments), is lower for individuals who experienced a transition towards higher depressive symptom levels during the study period versus those who did not.

## Materials and methods

### Sample

Our sample consisted of participants of the TRANS-ID Tapering study, a study that aimed at examining early warning signals of increases in depressive symptoms during and after tapering of antidepressant medication (for details, see refs. [28, 35]). In short, 69 individuals who had an earlier diagnosis with major depressive disorder (MDD) according to DSM-IV criteria monitored themselves for four months with weekly questionnaires, Ecological Momentary Assessment (EMA), actigraphy, and ECG sensors. These individuals made a shared decision with their mental health care provider to taper their antidepressant dosage (see Supplementary SA1 for details) and did not meet the criteria for MDD at baseline.

The study was approved by the Medical Ethical Committee of the University Medical Center Groningen (UMCG, METc2016.443). All patients were informed that they could stop their participation at any time and provided written informed consent prior to participation.

### Participants and procedures TRANS-ID tapering ECG sub-study

**Fig. 1:**
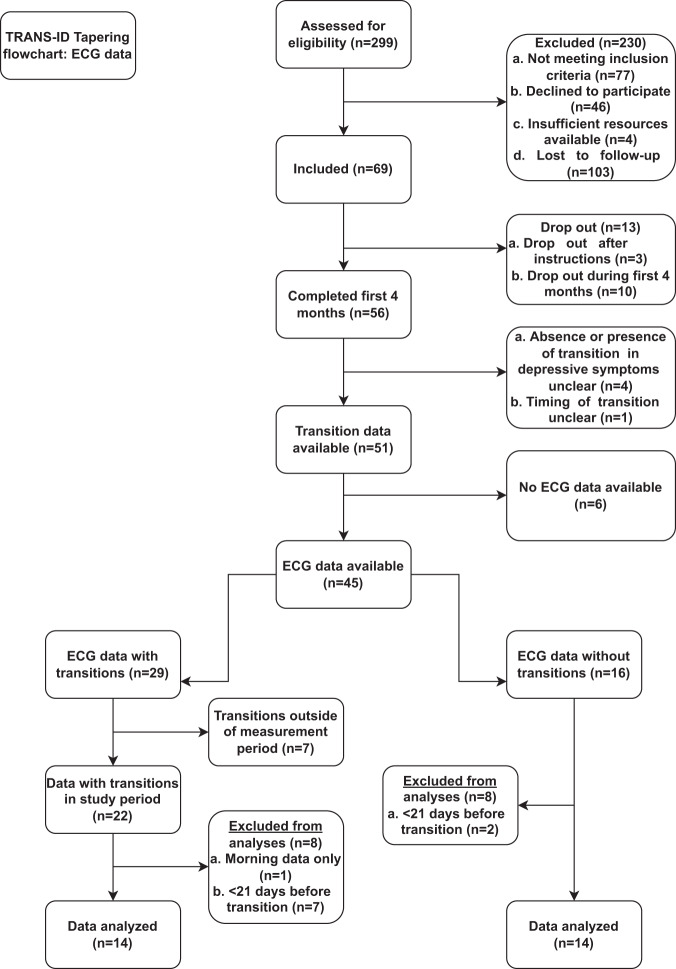
Flowchart describing the patient inclusion for the present study. At the end of various inclusion and exlusion criteria, 14 individuals with a transition and 14 individuals without were analyzed in the present study.

The flowchart of the TRANS-ID Tapering ECG sub-study is shown in Fig. 1. Out of 69 individuals, 50 individuals had usable ECG recordings. The presence or absence of transitions could be reliably calculated in 45 of these individuals. Among them, 29 experienced a transition in depressive symptoms, while 16 did not. From those with a transition, we excluded 7 individuals who did not have at least 3 weeks of data prior to the transition, to avoid spurious trends caused by too few datapoints. Also excluded from this group were 7 individuals whose transitions occurred outside of the ECG measurement period and 1 individual who only had morning assessments. Hence, of the original 69 individuals, valid ECG data were available for 14 individuals with a transition, which formed the transition group in the current paper. Of the 16 who did not show any transitions, 2 individuals were excluded, as they had less than 3 weeks of data available for analysis, leaving a sample of 14 individuals who did not experience any transitions for analysis.

#### ECG assessments and pre-processing

Participants performed their ECG assessments at home after receiving a 15-min step-by-step instruction on how to do so during the introductory session. Additionally, participants received a written manual (see https://osf.io/zbrxe/), ECG-electrodes, and contact details for 24/7 support (more details are given in Supplementary SA2). The ECG files were processed meticulously to extract the InterBeat-Interval (IBI) time-series data as detailed in Supplementary SA3.

### IBI data processing

#### Independent variables

Four quantifiers were derived from each IBI time-series assessment; the mean and standard deviation, and two complexity measures, namely the Higuchi dimension and the multiscale entropy. All four quantifiers have been widely used to study cardiac dynamics [15, 24, 36–39].

##### **Mean**

This is the average of the IBIs representing the mean time between two R-peaks. A higher mean IBI is reflective of a lower heart rate.

##### **Standard deviation**

The standard deviation of the IBI time series is referred to as the SDRR (standard deviation of RR intervals) and is a time-domain measure of heart rate variability [39].

##### **Higuchi dimension**

The Higuchi dimension estimates the fractal dimension of a time series directly, without any need for embedding in higher dimensions. Hence it can be reliably calculated even with fewer datapoints, as compared to some other estimates of the fractal dimension [40]. It calculates the scaling behavior of the length of the time-series curves when two parameters, namely the delay time and initial time, are varied [34]. In the present work, the maximum delay time is set to be 5, since the number of points used for calculating lengths is reduced at higher delays.

##### **Multiscale entropy**

The multiscale entropy measures the predictability of fluctuations in time series, at different scales of measurement. A higher value of entropy indicates a higher complexity of the time series. The multiscale entropy was estimated using the neurokit2 package [41] and the Higuchi dimension was calculated using the HDFA package [42] in Python v3.5.2.

#### Dependent variable

The dependent variable in the study was a transition towards higher levels of depression based on the following criteria: (1) a reliable increase (≥ 8.5 points) on the weekly assessed SCL-90 depression subscale, (2) persistence of this increase for at least three weeks, and/or start or increase in treatment, and/or interruption of tapering, (3) a meaningful increase in depressive symptoms as experienced by participants based on a consensus rating of emails, telephone calls, open text fields, and the evaluation interview (see also refs. [28, 43]).

For the analyses in this paper, in order to compare the samples of individuals who experienced a transition with those that did not, a pseudo-transition point was determined in the individuals who did not experience a transition. The transition times in the non-transitioning dataset were pair matched with the transition times of the transitioning dataset.

### Statistical analyses

The analyses are divided into within-subject and between-subject analyses, where the former identified changes in the quantifiers over time occurring within individuals, and the latter identified differences in average levels of the quantifiers during the 4 weeks of the study period between the individuals who experienced transitions and those who did not.

#### Within-individual analyses

To study changes at the level of an individual, each of the four quantifiers mentioned above was calculated for every IBI assessment, over a pre-transition period defined as 8 weeks before a transition. To avoid significant loss of data, all datasets were required to have a minimum of 3 weeks of data prior to the transition. These generated time series of quantifiers were categorized into the morning and evening time series. The Kendall correlation coefficient between these quantifiers and time was measured to determine the time trends. Significant time trends, as well as the direction of such trends preceding transitions towards greater depressive symptoms and in patients who stayed in remission were studied, and the number of individuals with significant trends were quantified.

#### Between-individuals analyses

To study mean differences between individuals who experienced a transition in future and those that did not, we first took the mean values for each quantifier per individual, by averaging over the value for each measurement through the baseline period. The baseline period was considered as the first 4 weeks of measurement, with a minimum requirement of at least 3 weeks of data. These averaged quantifiers were compared between the groups by using the non-parametric Mann–Whitney *U* test. In addition to having multiple advantages over the more commonly used t-test, the Mann–Whitney *U* test is more suitable for comparing small sample sizes and when the distributions are not normal, as in the case of heart rate variability measures [44, 45]. The effect size of the difference between the two groups was measured using Cohen’s *d* [46].

In addition, to study how well each quantifier predicted an upcoming transition, we used logistic regression models to predict presence versus absence of a future transition using the calculated quantifiers at baseline. Since ECG variables are known to depend significantly on age, we also tested the models with age added as a predictor. The goodness of fit was quantified using pseudo R^2^ values. The logistic regression was conducted in R version 3.6 [47].

Two sensitivity analyses were conducted on the data. First, since the actual time of transition from baseline varied between individuals, a sensitivity analysis was conducted by averaging over the whole pre-transition period identified for the individual-level study above. This controlled for the time elapsed between the assessments and the transition. The mean differences and predictive capabilities of the different quantifiers was then studied for the data averaged over the pre-transition period. A second analysis was conducted post hoc, to test whether the value of complexity parameters measured in the data arose from underlying nonlinear dynamics, or from stochasticity. For this, surrogate data were generated from the measured IBI data. The surrogates were generated using the Iterated Amplitude Adjusted Fourier Transform (IAAFT) method, which generated time-series with the same amplitude and Fourier spectrum as the original data but with randomized phases [48, 49]. The Higuchi dimension and multiscale entropy are then calculated for the surrogate data, and the distributions are compared with the original data. A significant difference between the distributions of quantifiers for data and surrogates indicate nonlinearity to be the cause of the observed values of complexity.

Dependence among the quantifiers at baseline was measured using the Spearman correlation coefficient (Spearman’s ρ) averaged at the level of an individual. Being a rank correlation coefficient Spearman’s ρ is both robust to outliers and can detect monotonic nonlinear trends. The *P* value for significance was set at 0.05. The Mann–Whitney *U* tests, Kendall and Spearman’s correlations were performed using the scipy package in Python version 3.5.2 [50]. All codes used for analysis are available at https://github.com/sgeorge91/TransID_IBI.

## Results

### Sample description

Data from 14 individuals who showed a transition during monitoring and data from 14 individuals who did not, were analyzed. The gender ratio did not significantly differ between the groups (78% versus 71% women in the transition and nontransition groups respectively, *P* = 0.66). Age was significantly higher in the transition group (*M* = 51.93 SD = 12.25, *t* = 2.19, *P* = 0.04) compared to the nontransition group (*M* = 41.79, SD = 11.30). The correlations between the different variables studied in our sample, averaged at baseline, with age, and with each other are listed in Table 1. The mean and standard deviation of the IBI time series in the morning was related with age. The highest correlations were observed between the evening mean and standard deviations, the morning entropy and morning dimension, and the evening mean and evening dimension measures.

**Table 1:** Correlations between the different ECG-derived quantifiers used in this study for the baseline assessments.

	Age	Mean_M_	SD_M_	HD_M_	MSE_M_
Age	1	**0.568**	**0.440**	−0.146	−0.096
Mean_E_	0.221	1 (0.188)	**0.646** (0.121)	−0.025 (0.037)	−0.238 (−0.193)
SD_E_	0.182	**0.855** (0.111)	1 (0.090)	−0.258 (−0.033)	0.084 (−0.144)
HD_E_	0.275	**0.709 (0.408)**	**0.634** (0.112)	1 (0.188)	**−0.742** (−0.361)
MSE_E_	−0.299	−0.091 **(−0.416)**	−0.003 (−0.072)	−**0.438** (−0.329)	**1 (0.540)**

*SD* standard deviation, *HD* Higuchi dimension, *MSE* multiscale entropy.

Entries above the diagonal represent correlations between the morning assessments and entries below denote correlations between the evening assessments. Correlations of the morning assessments with evening assessments are given between the parentheses. The table lists Spearman’s ρ correlations. Significant correlations (*P* < 0.05) are listed in bold.

### Within-individual analyses

We started by examining the IBI quantifiers for each individual for significant trends over time using Mann–Kendall trend test. Few trends were found in the hypothesized negative direction, that is, a decrease over time for the Higuchi dimension and the multiscale entropy. Within the morning assessments, we observe negative trends (14%) for the Higuchi dimension in 2 out of the 14 individuals who experienced a transition and no trends among those who did not. No negative trends were observed for any individual with or without a transition in depression for the entropy quantifier. For the evening assessments, no negative trends were found for the Higuchi dimension, whereas one negative trend was found an individual without a transition (7%). No negative trends were observed for any of the individuals in either group for the entropy quantifier.

Positive time trends were found in the morning assessments in three individuals (21%) for the Higuchi dimension and in 1 individual (7%) for the entropy among those with a transition. Three individuals in the non-transitioning group (21%) showed positive trends too for the Higuchi dimension, whereas no individuals in the non-transitioning group showed any trend for the entropy. In the evening assessments, the transitioning group showed no positive trends for the Higuchi dimension, whereas the entropy showed positive trends in two individuals (14%). The non-transitioning group showed positive trends in four individuals (29%) for the Higuchi dimension, and no trends for entropy. Detailed results for the within-individual analyses showing the trends for each individual and quantifier are presented in Supplementary SA4.

### Between-individuals analyses

**Fig. 2:**
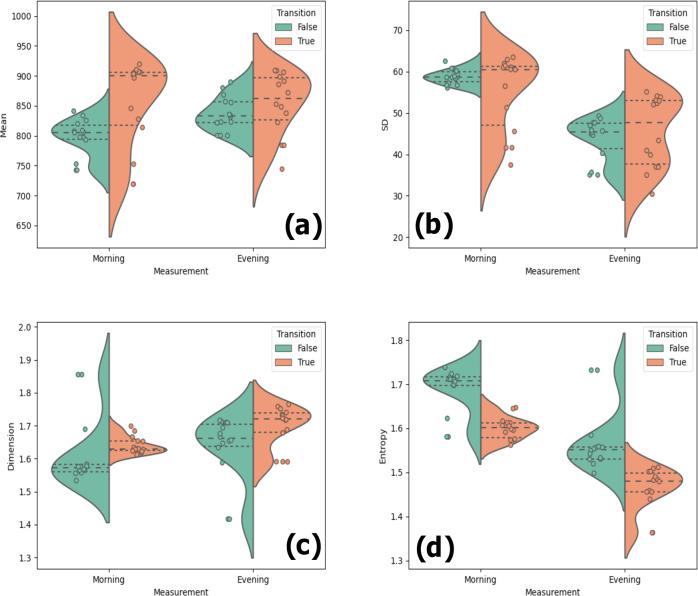
Violin plots showing the differences in the distributions of the person-averaged quantifiers between individuals who experienced a transition and those who did not. The panels show **a** mean, **b** standard deviation, **c** Higuchi dimension, and **d** Multiscale entropy. The distribution for individuals who experienced a transition are in orange and those who did not are in green. The circles represent the quantifier values for each individual, scattered randomly along the x-axis. The quantifiers were averaged over the baseline periods.

Next, we studied group differences in the quantifiers averaged within individuals over the baseline period of 4 weeks. The mean differences for these averaged quantifiers between individuals who experienced a transition, and those who did not are listed in Table 2. Figure 2 shows the corresponding distributions for the two groups as violin plots. For the morning assessments, individuals who experienced transitions showed a significantly higher mean IBI and Higuchi dimension, and a significantly lower entropy than individuals who did not experience a transition. For the evening assessments, the individuals who experienced a transition showed a significantly higher Higuchi dimension and a significantly lower entropy than individuals who did not experience a transition.

**Table 2:** Differences in the person-averaged quantifiers between the group which experienced a transition and the group that did not.

Quantifier	Transition group (M ± SD)	Non-transitioning group (M ± SD)	*z*-score	*P* value	Cohen’s *d*
**Mean**_**M**_	**852.161** ± **71.537**	**797.921** ± **30.415**	**2.412**	0.018*	**0.987**
SD_M_	54.743 ± 8.976	58.788 ± 1.752	0.253	0.133	−0.625
**HD**_**M**_	**1.641** ± **0.025**	**1.615** ± **0.103**	**2.642**	0.009**	**0.344**
**MSE**_**M**_	**1.600** ± **0.025**	**1.687** ± **0.050**	**−3.469**	<0.001***	**−2.185**
Mean_E_	853.034 ± 51.104	837.026 ± 28.159	1.309	0.334	0.388
SD_E_	45.475 ± 8.362	43.738 ± 4.907	0.804	0.525	0.253
**HD**_**E**_	**1.698** ± **0.061**	**1.635** ± **0.096**	**2.550**	0.012*	**0.785**
**MSE**_**E**_	**1.464** ± **0.046**	**1.569** ± **0.069**	**−4.296**	<0.001***	**−1.797**

*SD* standard deviation, *HD* Higuchi dimension, *MSE* multiscale entropy.

The quantifiers, namely the mean, standard deviation, Higuchi dimension, and Multiscale entropy, were calculated from IBI measurements. The assessments were taken every day and averaged over the baseline period. The subscripts (M or E) refer to the time of the day when the ECG measurement was carried out (morning or evening). The differences between the groups were measured using a Mann–Whitney *U* test. Results in bold with asterisk indicate significant differences; *P <0.05, **P <0.01, ***P <0.001.

The results of the logistic regression model used to predict whether the individual will undergo a transition or not are given in Table 3. The model where the entropy alone predicts the transitions stood out with the highest explained variance among all the models considered, with a lower baseline entropy significantly predicting a future transition towards higher depressive symptom levels.

**Table 3:** Logistic regression models predicting depressive transitions during study period^a^.

Predictor	Estimate	SE	*z*-value	*P* value	*R*^2^	Correctly predicted (%)
Transition~Age						
Age	**0.069**	**0.035**	**1.967**	0.049*	0.203	67.9
Transition~Mean_X_						
Mean_M_	**16.803**	**7.774**	**2.161**	0.031*	0.253	75.0
Mean_E_	9.340	9.407	0.993	0.321	0.048	64.3
Transition~SD_X_						
SD_M_	−103.945	71.873	−1.446	0.148	0.122	60.7
SD_E_	36.755	55.556	0.662	0.508	0.021	39.3
Transition~HD_X_						
HD_M_	4.706	5.407	0.870	0.384	0.039	75.0
HD_E_	11.529	6.769	1.703	0.089	0.191	67.9
Transition~MSE_X_						
MSE_M_	**−37.75**	**12.49**	**−3.021**	0.002**	0.635	82.1
MSE_E_	**−150.73**	**73.41**	**−2.053**	0.040*	0.880	96.4

^a^The models show how well transition status is predicted by the baseline quantifiers. The listed *R*^2^ value is the Nagelkerke *R*^2^. Results in bold with asterisk indicate significant differences; *P <0.05, **P < 0.01.

**Fig. 3:**
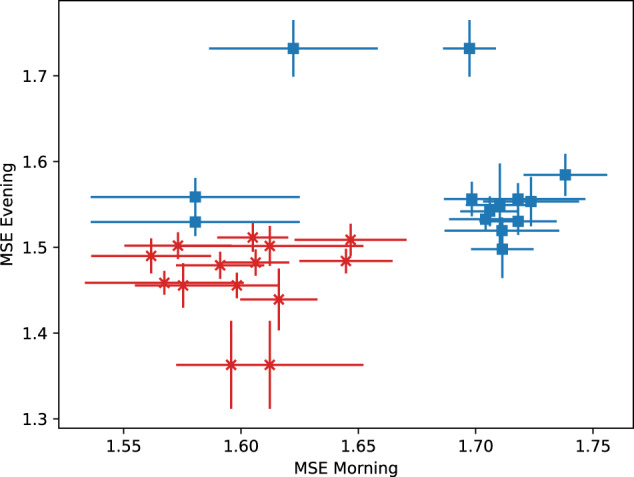
Scatter plot showing the entropy values for the morning and evening for each individual. Red crosses represent individuals who experienced a transition, and the blue squares represent individuals who did not. The error bars represent the standard error.

In Fig. 3, we show the values of baseline entropy and error for each individual, calculated using the morning and evening assessments. The individuals with a transition occupy a region in the lower left of the graph, pointing out once again that low values of entropies were largely associated with individuals who experienced transitions in the study period. A grid search on the entropy values found 1.67 as the morning entropy value (25/28 individuals correctly classified) and 1.51 as the evening entropy value that best discriminates the two groups (27/28 individuals correctly classified). Noticeably, in this sample, a smaller within-person standard deviation was observed in the entropies associated with the evening assessments, indicating that the entropy measurements during the morning were less stable than in the evening.

### Sensitivity analyses

The first sensitivity analysis, calculating the correlations, mean differences, and predictive capacity of the quantifiers averaged over the pre-transition period instead of the baseline period, are presented in the supplements (Supplementary SA5). Again, lower entropy over the pre-transition period was most strongly associated with the presence of a future transition in depressive symptoms, showing the highest significance in the Mann–Whitney *U* test and highest explained variance in a logistic regression.

The second analysis calculated the deviations of the complexity quantifiers considered in the study from surrogate data. The details of the analysis are presented in the supplements (Supplementary SA6). We find significant differences (*P* < 0.05) between the distributions of both the Higuchi dimension and the multiscale entropy quantified from the original time-series data and surrogates. In particular, the multiscale entropy of the surrogate time-series data showed highly significant (*P* < 0.001) deviations from the original time-series.

## Discussion

This work explored how the complexity of IBI time-series data behaves before a transition toward more severe depressive symptoms. While very few trends within individuals over time were observed in the different cardiac quantifiers before a transition toward depression, we found that the baseline levels of entropy were significantly lower for individuals who experienced a transition compared to individuals who did not. In addition, we found higher mean IBIs and higher Higuchi dimension for individuals who experienced transitions. This seems to indicate that below a threshold level of complexity of cardiac dynamics, individuals who taper antidepressants are vulnerable for recurrence. The combination of higher fractal dimension and lower entropy in individuals who experienced transitions suggests that these time series exhibit more noisy behavior [51, 52]. There is large variability in the values of complexity variables reported in literature, due to the large heterogeneity in the parameter values used in the estimating algorithm. However, the values reported previously in the literature for the Higuchi dimension and multiscale entropy are in line with values found in the current study [53–55]. In addition, the values of the complexity measures showed a significant difference from surrogate datasets, suggesting that the obtained measures of complexity arise from deterministic nonlinearity and not from randomness.

The present work is significant in multiple ways. First, it provides little evidence to support the existence of within-individual warning signals in IBI time-series data that precede and predict an upcoming depressive transition, in line with similar studies using ecological momentary assessment and actigraphy data [27, 43]. Second, the current study shows that lower entropy values derived from IBI time series indicate that individuals are more likely to experience an increase in depressive symptoms in the coming months, which may be helpful information when deciding on whether antidepressant medication should be tapered. Based on this we speculate that IBI time-series derived entropy quantifiers could become promising biomarkers for determining if antidepressants can be tapered with a reduced risk of recurrence of depression. Third, the current study answers an important question on how the complexity and variability of cardiac dynamics change before the recurrence of depression. While we find that the complexity of cardiac dynamics is significantly lower in individuals who experienced a transition towards increased depressive symptoms, no decrease in complexity over time was observed before transitions within most individuals. An explanation for the absence of this change is that the decrease in complexity may have taken place at a timescale longer than the 8 weeks considered in the present study. An alternative reason could be that decreased complexity is a stable vulnerability that persisted in some individuals from a previous episode of depression, since the sample consisted of individuals who experienced an episode previously. Based on past work, we expect that an earlier episode would have been associated with decreased complexity of cardiac dynamics [11, 17], and individuals who experienced a transition towards higher depressive symptoms in this sample possibly did not fully recover their complexity [56].

A major limitation of the study, originally designed for within-individual analysis, is the small sizes of the groups for between-subject analyses. The current study explored multiple indicators in this small group of participants and may therefore be overfitting the sample. Moreover, despite the large effect sizes observed for the entropy, inter-individual differences may not be fully captured. A second limitation is the tapering of antidepressants in the current sample. The cardiotropic effects of antidepressant medication on the dynamics of the heart are well documented, with many of them causing a reduction in the mean heart rate and its variability [24, 57–59]. This intake could dominate the effects of the upcoming transition, if any, on the cardiac dynamics. A considerable fraction of the present sample majorly tapered their antidepressants during the baseline period (17 participants reported tapering more than 2/3rds of their dosage in the baseline period). This could have changed the cardiac dynamics during the baseline period in both groups. A third limitation was the strict protocols set for the self-assessment of ECG. Apart from being burdensome, such strict assessment instructions could have resulted in cardiac dynamic signals with less noise, limiting generalization to other studies with ambulatory ECG assessments. However, with improvements in ambulatory ECG monitoring using less obstructive devices, it may be possible to monitor the complexity of the heart more easily for an extended time in normal daily life [60, 61]. Future studies are needed to estimate how well the results of the current study generalize to new samples.

The promising results of our study points to the need for a larger exploration of the use of cardiac complexity measures as a predictor for depression. If validated by future studies, patients who are planning on tapering their antidepressant medication may assess their ECG at home to assist decision-making [62]. Furthermore, we suggest including the IBI complexity measures used in this study in other models which predict the recurrence of depression [63, 64]. While this study finds that complexity measures are lower in individuals who experience a recurrence of depressive symptoms, the results do not indicate whether this is true in individuals who experience a depressive episode for the first time, or in individuals who are not tapering antidepressant medication. We recommend exploring the variation of complexity of cardiac dynamics prior to transitions towards depression in other samples, where these drawbacks may not exist.

In conclusion, this study suggests that quantifiers of complexity of cardiac dynamics can serve as an indicator for future recurrence of depressive transitions. While the study failed to find any trends in these quantifiers preceding depressive symptom transitions, it suggests a strong possibility of using complexity-based quantifiers to identify individuals at risk for recurrence of depression [65]. Though many challenges remain to be solved before a clinical implementation is feasible, we believe that these indicators can greatly aid in decision-making in the context of tapering antidepressants.

## Supplementary information

Supplementary material: Using complexity of cardiac dynamics as a predictor of recurrence of depression in individuals tapering their antidepressants use
