# Supplementary material for: Predicting recurrence of depression using cardiac complexity in individuals tapering antidepressants
Source: Transl Psychiatry. 2023 May 30;13:182. doi: 10.1038/s41398-023-02474-7 (PMC10229565; doi:10.1038/s41398-023-02474-7)
Supplement: Supplementary file 1 — Supplementary material: Using complexity of cardiac dynamics as a predictor of recurrence of depression in individuals tapering their antidepressants use [file 41398_2023_2474_MOESM1_ESM.docx]

**Supplementary material: Using complexity of cardiac dynamics as a predictor of recurrence of depression in individuals tapering their antidepressants use**

**Authors:** Sandip V. George, PhD^1,2^, Yoram K. Kunkels, MSc^1^, Arnout Smit, PhD^1^, Marieke Wichers, PhD^1^, Evelien Snippe, PhD^1^, Arie M van Roon, PhD^3^, Harriëtte Riese, PhD^1^

**Affiliation:** ^1^University of Groningen, University Medical Center Groningen, Department of Psychiatry, Interdisciplinary Center Psychopathology and Emotion regulation (ICPE), Groningen, The Netherlands

^2^ University College London, Department of Computer Science, London, United Kingdom

^3^University of Groningen, University Medical Center Groningen, Department of Vascular Medicine, Groningen, The Netherlands

Contents

[Supplementary SA1: Details of medication use 3](#_Toc130427310)

[Supplementary SA2: ECG assessment procedure 4](#_Toc130427311)

[Supplementary SA3: ECG data preprocessing 5](#_Toc130427312)

[Supplementary SA4: Within subject analysis 6](#_Toc130427313)

[Supplementary SA5: Pre-transition period 9](#_Toc130427314)

[Supplementary SA6: Surrogate analysis 14](#_Toc130427315)

[**References** 15](#_Toc130427316)

# Supplementary SA1: Details of medication use

In this supplementary we broadly describe the kinds of medication used in the two groups. In table S1.1 we lay down the number of patients who took each type of anti-depressants in the group which experienced transitions and the group that did not, along with the effect of each on the blood pressure and heart rate(1–7).

**Table ST1.1:** Number of patients who used particular types of medication among the group which experienced transitions and the group which did not

| **Medication** | **Transition group (n)** | **Non transition group (n)** |
| --- | --- | --- |
| Venlafaxine (2) | 5 | 6 |
| Setraline (1) | 3 | 1 |
| Paroxetine (1) | 3 | 1 |
| Flouxitine (1) | 3 | 1 |
| Bupropion (2) | 1 | 1 |
| Escilatopram(1) | 1 | 0 |
| Citalopram (1) | 0 | 3 |
| Trimipramine (-1) | 1 | 0 |
| Clomipramine (-1) | 0 | 1 |

**Note:** . Since some of the patients in the transition group took more than one medication, the total is more than the size of the group (14).

(2) Minor effect on blood pressure (increase) and HRV.

(1) No effect on blood pressure HRV expected.

(-1) Minor effect on blood pressure (decrease), orthostatic hypotension can occur.

# Supplementary SA2: ECG assessment procedure

Participants were invited to a lab for a two-hour introduction session in which they participated in a clinical interview and received instructions about the monitoring procedures for assessment of all measures (for details see (8)). Instructions for the 3-lead *Cortrium* ECG sensor (9) monitoring took 10-15 minutes. Participants were instructed how to attach the *Cortrium* to their chest with 3M Red Dot pre-gelled ECG-electrodes. The *Cortrium* software was installed on their smartphone to make a Bluetooth connection with the sensor and participants were familiarized with the data upload procedure. Instructions includes opening the *Cortrium* app to make sure the ECG signal is visible on all three leads, checking whether valid R-peaks are visible in the ECG recording, and how to initiate recording and uploading the ECG assessment to the secured UMCG server. Sample rate was 250 Hz while a backup of the data was also saved on the smartphone. Participants thus received a step-by-step instruction on how to take the ECG assessments at home. Participants were to repeat the heart rate measurement without help to ensure they were able to correctly perform the assessments themselves. They also received a written manual (see https://osf.io/zbrxe/), ECG-electrodes, and contact details for 24/7 support.

For four months, ECGs were assessed two times a day after the first and fifth (i.e. last) ESM measurement (10) which corresponded to the morning and evening measurements respectively. Participants were instructed to sit down when filling-out their ESM. Filling out the ESM took between 5 and 10 minutes thereby giving the heart the opportunity to signal a stationary, rest ECG (8). After filling-out the ESM questionnaire, participants attached the ECG sensor to their chest. Participants were instructed not to talk during the 5 minutes recording in sitting posture to standardize recording and prevent activity and posture bias in the ECG recordings (8). The researchers regularly checked the quality of the raw ECG data and contacted the participant for further instructions if violations of the recording protocol were suspected.

# Supplementary SA3: ECG data preprocessing

Data pre-processing steps included converting raw *Cortrium* device output files, checking file integrity, R-peak triggering, and correcting for (motion) artefacts. *PreCARSPAN* version 3.83 (11) was used for pre-processing the ECG-data conform an in-house protocol. This involved R-peak triggering and artifact correction to obtain InterBeat Interval (IBI, in ms) time-series data. In *PreCARSPAN*, missing data were interpolated up to a maximum of 10 sec. but in not more than 10% of the total block duration. Otherwise, time-series data in a block was set to missing due to poor data quality. 1% was due to participant error, 2.1% due to movement artifacts, 10.1% due to participants not finishing the measurements, 11.5% were excluded due to incomplete data and 74.6% were excluded due to other technical difficulties. Data analysts were first trained by analyzing ten example files under supervision of an expert ECG data analyst. Data analysts were allowed to work on the real time-series data files after sufficiently high intraclass correlation coefficient (ICC) values (ICC > 0.95) between the training files processed by the analyst and those processed by the expert cardiology analyst were attained.

It was checked whether the data were not too noisy for analysis, whether the R-squared values between the IBI time series and time were less than 0.30, and whether the variation coefficient of IBI values were above 20%. 1.03% of the raw IBI files were found to exceed these criteria. When such physiologically implausible values were detected, these were followed-up up with an additional check in the raw data to make sure no R-peaks or artefacts were missed during data pre-processing.

# Supplementary SA4: Within subject analysis

In this supplementary we provide additional information on the within-subject analysis described in the main text. In Table ST5.1 we present the values of the Kendall correlation coefficient for the individuals in the study.

Table ST4.1: Trends for each quantifier within individuals over time.

|  | **Mean_M_** | **SD_M_** | **Dimension_M_** | **Entropy_M_** | **Mean_E_** | **SD_E_** | **Dimension_E_** | **Entropy_E_** |
| --- | --- | --- | --- | --- | --- | --- | --- | --- |
| *T01* | -0.16 | -0.08 | -0.04 | -0.02 | -0.15 | -0.03 | -0.03 | 0.15 |
| *T02* | 0.14 | 0.18 | 0.12 | 0.04 | -0.06 | 0.00 | -0.12 | 0.09 |
| *T03* | **-0.25** | -0.08 | **-0.22** | 0.10 | 0.20 | **0.27** | 0.10 | 0.04 |
| *T04* | 0.28 | 0.21 | 0.23 | -0.04 | 0.22 | 0.16 | 0.17 | 0.18 |
| *T05* | 0.01 | 0.07 | **0.24** | 0.10 | -0.16 | -0.05 | 0.04 | -0.03 |
| *T06* | 0.13 | 0.21 | 0.13 | 0.08 | **0.22** | **0.40** | -0.20 | -0.01 |
| *T07* | -0.03 | -0.03 | -0.13 | 0.05 | -0.08 | 0.08 | -0.12 | 0.08 |
| *T08* | 0.22 | 0.16 | -0.01 | 0.10 | -0.01 | -0.07 | 0.15 | -0.07 |
| *T09* | -0.05 | **0.29** | 0.09 | 0.22 | 0.09 | -0.09 | 0.21 | 0.07 |
| *T10* | **-0.29** | 0.01 | **-0.26** | **0.33** | -0.13 | -0.08 | -0.03 | **0.29** |
| *T11* | 0.058 | **0.25** | **0.38** | 0.11 | -0.12 | 0.00 | 0.05 | -0.04 |
| *T12* | 0.02 | 0.05 | **0.21** | -0.12 | 0.05 | 0.03 | 0.14 | 0.08 |
| *T13* | -0.05 | 0.02 | -0.16 | 0.15 | **0.30** | 0.09 | -0.10 | **0.19** |
| *T14* | 0.04 | -0.03 | 0.14 | -0.04 | -0.02 | -0.07 | 0.14 | 0.13 |
| *N01* | -0.03 | -0.08 | 0.16 | 0.03 | 0.00 | 0.05 | 0.12 | -0.06 |
| *N02* | 0.03 | 0.13 | 0.08 | 0.17 | -0.05 | 0.09 | 0.11 | 0.05 |
| *N03* | **0.44** | **0.31** | **0.47** | -0.04 | 0.09 | 0.10 | 0.18 | 0.00 |
| *N04* | 0.02 | 0.19 | 0.19 | 0.15 | 0.19 | 0.06 | **0.41** | 0.21 |
| *N05* | **0.51** | **0.29** | **0.25** | 0.02 | **0.42** | 0.07 | **0.35** | 0.11 |
| *N06* | -0.10 | -0.16 | 0.05 | 0.21 | -0.18 | **-0.25** | 0.02 | 0.02 |
| *N07* | -0.05 | -0.15 | **0.32** | -0.07 | **-0.24** | -0.10 | **0.27** | -0.10 |
| *N08* | **-0.34** | 0.03 | -0.16 | 0.12 | -0.15 | 0.10 | **-0.25** | 0.04 |
| *N09* | -0.03 | 0.04 | 0.18 | 0.10 | -0.05 | -0.03 | 0.12 | 0.11 |
| *N10* | -0.17 | 0.05 | 0.17 | -0.07 | -0.19 | -0.04 | 0.20 | 0.01 |
| *N11* | -0.11 | -0.09 | 0.13 | 0.12 | **0.32** | 0.17 | **0.35** | -0.05 |
| *N12* | 0.06 | 0.37 | -0.01 | -0.18 | 0.00 | 0.17 | -0.19 | 0.14 |
| *N13* | 0.12 | 0.18 | 0.07 | -0.04 | **0.28** | **0.34** | 0.13 | 0.05 |
| *N14* | -0.20 | -0.02 | -0.22 | 0.04 | 0.14 | -0.11 | -0.01 | 0.12 |

**Note**: The significant time trends (p<.05) are shown in bold. The subscripts (M or E) refer to the time of the day when the ECG measurement was carried out (morning or evening). The participants with T are the ones who experienced a transition and NT are the ones who did not.

# Supplementary SA5: Pre-transition period

In this supplementary we present the differences in the mean levels and predictive capacities of the measures averaged over the pre-transition period, within an individual. The pre-transition period is defined as 4 to 8 weeks prior to a transition period. Unlike the baseline, the quantifiers averaged over the pre transition period represent the measured variables immediately prior to the point when the transition occurred.

We first check for the correlations between the various quantifiers, as well of the quantifiers with age. This is listed in Table ST 5.1. We then use a Mann Whitney u-test to compare the distributions of the quantifiers between individuals who experienced a transition and those who did not. Table ST 5.2 lists the differences between the two groups for these person-averaged measurements. Figure SF 5.1 represents the corresponding violin plots showing the distributions of the quantifiers across individuals as averaged over the pre transition period. All quantifiers, except the standard deviation, are seen to be significantly different for the evening measurements, with the mean and dimension having significantly higher values, and the entropy showed significantly lower for individuals who experienced transitions compared who individuals who did not. Only the dimension and entropy show significantly lower values for individuals who experienced transitions, in the morning measurements.

Next, we use a logistic regression model to analyze how well each of the quantifiers in this study predicts a depressive transition in future. Table ST 5.3 lists the results of the logistic regression for different models. The logistic regression models showed that higher levels of age and evening measurements of the mean significantly predicted being in the transition group, whereas lower levels of entropy significantly predicted being in the transition group. Since the age significantly correlated with mean, we checked for how well the mean predicted transitions when controlling for age. The mean continued to significantly predict a depressive transition in future. The simplest model with the best fit is again shown to the transitions predicted using the entropy alone, predicting over 80% of transitions for both morning and evening measurements.

Table ST5.1: Correlations between the different quantifiers used in the study.

|  | Age | Mean_M_ | SD_M_ | HD_M_ | MSE_M_ |
| --- | --- | --- | --- | --- | --- |
| Age | 1 | **0.436** | 0.235 | -0.029 | 0.076 |
| Mean_E_ | 0.243 | 1 | **0.787** | 0.187 | -0.055 |
| SD_E_ | 0.183 | **0.741** | 1 | 0.091 | -0.043 |
| HD_E_ | 0.294 | **0.829** | **0.728** | 1 | **-0.823** |
| MSE_E_ | -0.199 | -0.227 | -0.039 | -**0.385** | 1 |

**Note:** Entries above the diagonal represent the morning measurements and the entries below denote the evening measurements. The table lists the Spearman’s ρ. Significant correlations (p<.05) are listed in bold

Table ST5.2: Differences in the person-averaged mean, standard deviation, Higuchi dimension and Multiscale entropy from IBI measurements, between the group which experienced a transition and the group that did not.

| Quantifier | Transition group | Non transitioning group | z-statistic | p-value | Cohen’s d |
| --- | --- | --- | --- | --- | --- |
| Mean_M_ | 835.2±74.3 | 791.8±35.7 | 1.815 | 0.077 | 0.746 |
| SD_M_ | 55.0±9.9 | 57.5 ±3.5 | 0.666 | 0.534 | 0.337 |
| HD_M_ | **1.60±0.03** | **1.59±0.11** | **2.228** | **0.029*** | **0.086** |
| **MSE_M_** | **1.62±0.02** | **1.67±0.06** | **- 2.688** | **0.007 **** | **-1.097** |
| **Mean_E_** | **856.7±52.2** | **815.7±18.5** | **2.367** | **0.021*** | **1.020** |
| SD_E_ | 48.2±10.6 | 42.0±3.3 | 1.677 | 0.103 | 0.837 |
| **HD_E_** | **1.71±0.06** | **1.63±0.10** | **3.010** | **0.003**** | **0.961** |
| **MSE_E_** | **1.47±0.05** | **1.56±0.08** | **-4.020** | **<0.001***** | **-1.434** |

***Note:*** The measurements were taken every day and averaged over the pre-transition period. The subscripts (M or E) refer to the time of the day when the ECG measurement was carried out (morning or evening). SD = Standard Deviation HD =Higuchi Dimension, MSE=Multiscale Entropy. Results in bold with asterisk indicate significant differences; p<.05 *, p<.01 **, p<.001***

Table ST5.3: Results of a logistic regression model showing how well transition status is predicted by the pre transition period quantifiers.

| Predictor | Estimate | SE | z-value | p-value | R^2^ | Correctly predicted % |
| --- | --- | --- | --- | --- | --- | --- |
| Transition~Age | | | | |  |  |
| Age | **0.069** | **0.035** | **1.967** | **0.049*** | 0.203 | 67.9 |
| Transition~Mean | | | | | |  |
| Mean_M_ | 12.457 | 7.099 | 1.755 | 0.080 | 0.161 | 71.4 |
| Mean_E_ | **24.850** | **11.286** | **2.202** | **0.028*** | 0.269 | 75.0 |
| Transition~Age+Mean | | | | | |  |
| Age | 0.048 | 0.036 | 1.331 | 0.183 |  |  |
| Mean_M_ | 9.267 | 7.477 | 1.239 | 0.215 | 0.237 | 57.1 |
| Age | 0.065 | 0.039 | 1.657 | 0.098 |  |  |
| Mean_E_ | **22.244** | **11.125** | **1.999** | **0.046*** | 0.381 | 67.9 |
| Transition~SD | | | | | |  |
| SD_M_ | -45.641 | 52.746 | -0.865 | 0.387 | 0.037 | 57.1 |
| SD_E_ | 108.310 | 56.746 | 1.909 | 0.056 | 0.195 | 75.0 |
| Transition~Dimension | | | | | |  |
| Dimension_M_ | 1.101 | 4.828 | 0.228 | 0.820 | 0.003 | 67.9 |
| Dimension_E_ | 14.324 | 7.344 | 1.951 | 0.051 | 0.278 | 67.9 |
| Transition~MSE | | | | | |  |
| Entropy_M_ | **-21.833** | **9.321** | **-2.342** | **0.019*** | 0.298 | 82.1 |
| Entropy_E_ | **-129.01** | **57.10** | **-2.259** | **0.024*** | 0.765 | 85.7 |

**Note:** The listed R^2^ value is the Nagelkerke R^2^. The subscripts (M or E) refer to the time of the day when the ECG measurement was carried out (morning or evening). The quantifiers were averaged over the pre transition periods. Results in bold with asterisk indicate significant differences; p<.05 *, p<.01 **, p<.001***

Figure SF5.1: Violin plots showing the differences in the distributions of the person averaged (a) mean (b) standard deviation (c) Higuchi dimension and (d) Multiscale entropy between individuals who experienced a transition (Orange) and those that did not (Green).


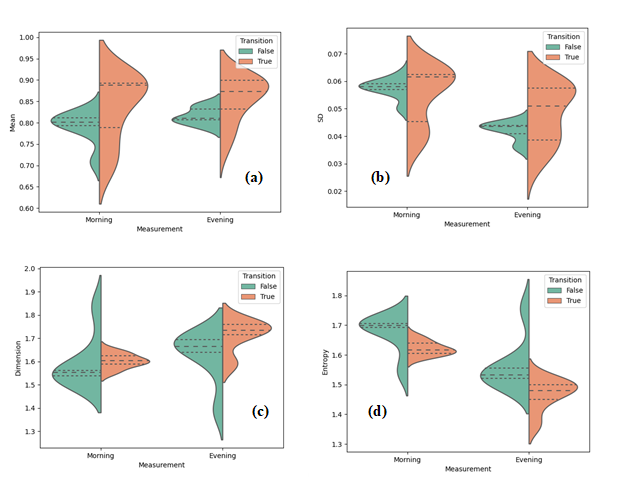


# Supplementary SA6: Surrogate analysis

In this supplementary we present the results of the analysis conducted on surrogate data derived from the IBI time series. The surrogate data was generated from the original data through the method of Iterated Amplitude Adjusted Fourier Transform (IAAFT), which preserved the amplitude and power spectrum of the original data, while randomizing the phases(12). In effect, this generates surrogate copies that are linear stochastic processes, while preserving the periodicities and amplitude spectrum present in the original data. In our analysis, 10 surrogate datasets were generated for each of the morning and evening IBI time series, resulting in about 280 surrogate datasets for each individual. The Higuchi dimension and multiscale entropy are then calculated for each surrogate dataset, and averaged over all surrogates for an individual. Differences between the distributions of the original data and surrogates are measured using the Mann-Whitney u test.

Table ST6.2: Differences in the person-averaged Higuchi dimension and Multiscale entropy from IBI measurements, between the original data and the surrogates.

| Quantifier | Original Data | Surrogate data | z-statistic | p-value |
| --- | --- | --- | --- | --- |
| HD_M_ | **1.58±0.14** | **1.65±0.10** | **2.228** | **0.006**** |
| **MSE_M_** | **1.65±0.13** | **1.42±0.14** | **- 2.688** | **<0.001***** |
| **HD_E_** | **1.73±0.17** | **1.74±0.10** | **3.010** | **0.003**** |
| **MSE_E_** | **1.49±0.13** | **1.26±0.13** | **-4.020** | **<0.001**** |

***Note:*** The measurements were taken every day and averaged over the baseline period. The subscripts (M or E) refer to the time of the day when the ECG measurement was carried out (morning or evening). HD =Higuchi Dimension, MSE=Multiscale Entropy. Results in bold with asterisk indicate significant differences; p<.05 *, p<.01 **, p<.001***

# **References**

1. Guo MY, Etminan M, Procyshyn RM, Kim DD, Samii A, Kezouh A, et al. Association of Antidepressant Use with Drug-Related Extrapyramidal Symptoms: A Pharmacoepidemiological Study. J Clin Psychopharmacol. 2018;38(4).

2. Haddad PM, Dursun SM. Neurological complications of psychiatric drugs: Clinical features and management. Vol. 23, Human Psychopharmacology. 2008.

3. Petry N, Lupu R, Gohar A, Larson EA, Peterson C, Williams V, et al. CYP2C19 genotype, physician prescribing pattern, and risk for long QT on serotonin selective reuptake inhibitors. Pharmacogenomics. 2019;20(5).

4. Jasiak NM, Bostwick JR. Risk of QT/QTc Prolongation Among Newer Non-SSRI Antidepressants. Vol. 48, Annals of Pharmacotherapy. 2014.

5. Fava GA, Benasi G, Lucente M, Offidani E, Cosci F, Guidi J. Withdrawal symptoms after serotonin-noradrenaline reuptake inhibitor discontinuation: Systematic review. Vol. 87, Psychotherapy and Psychosomatics. 2018.

6. Stahl’s Essential Psychopharmacology: Prescriber’s Guide (5th edition). Reference Reviews. 2015;29(1).

7. Fernandez A, Bang SE, Srivathsan K, Vieweg WVR. Cardiovascular side effects of newer antidepressants. Vol. 7, Anadolu Kardiyoloji Dergisi. 2007.

8. Kunkels YK, van Roon AM, Wichers M, Riese H. Cross-instrument feasibility, validity, and reproducibility of wireless heart rate monitors: Novel opportunities for extended daily life monitoring. Psychophysiology. 2021;58(10).

9. Cortrium. https://www.cortrium.com. 2019.

10. Smit AC, Snippe E, Hoenders HJR, Wichers M. Transitions In Depression: If, how, and when depressive symptoms increase during and after tapering of antidepressant medication. Submitted. 2020;

11. Greaves-Lord K, Tulen J, Dietrich A, Sondeijker F, van Roon A, Oldehinkel A, et al. Reduced autonomic flexibility as a predictor for future anxiety in girls from the general population: The TRAILS study. Psychiatry Res. 2010;179(2).

12. Schreiber T, Schmitz A. Improved surrogate data for nonlinearity tests. Phys Rev Lett. 1996;77(4).
